# A Feasibility Open-Labeled Clinical Trial Using a Second-Generation Artificial-Intelligence-Based Therapeutic Regimen in Patients with Gaucher Disease Treated with Enzyme Replacement Therapy

**DOI:** 10.3390/jcm13113325

**Published:** 2024-06-05

**Authors:** Noa Hurvitz, Tama Dinur, Shoshana Revel-Vilk, Samuel Agus, Marc Berg, Ari Zimran, Yaron Ilan

**Affiliations:** 1 Departments of Medicine and Neurology, Hadassah Medical Center, Jerusalem 9112001, Israel; noa.hurvitz@mail.huji.ac.il; 2 Gaucher Unit, The Eisenberg R&D Authority, Shaare Zedek Medical Center, Jerusalem 9103102, Israel; dinurtama@gmail.com (T.D.); srevelvilk@gmail.com (S.R.-V.); azimran@gmail.com (A.Z.); 3 Faculty of Medicine, Hebrew University, Jerusalem 9112001, Israel; 4 Oberon Sciences and Area 9 Innovation, Chestnut Hill, MA 02467, USA; sam.agus@samagus.com (S.A.); marc@area9.dk (M.B.); 5 Stanford University, Palo Alto, CA 94305, USA

**Keywords:** Gaucher Disease, enzyme replacement therapy, artificial intelligence

## Abstract

**Background/Objectives:** Gaucher Disease type 1 (GD1) is a recessively inherited lysosomal storage disorder caused by a deficiency in the enzyme β-glucocerebrosidase. Enzyme replacement therapy (ERT) has become the standard of care for patients with GD. However, over 10% of patients experience an incomplete response or partial loss of response to ERT, necessitating the exploration of alternative approaches to enhance treatment outcomes. The present feasibility study aimed to determine the feasibility of using a second-generation artificial intelligence (AI) system that introduces variability into dosing regimens for ERT to improve the response to treatment and potentially overcome the partial loss of response to the enzyme. **Methods:** This was an open-label, prospective, single-center proof-of-concept study. Five patients with GD1 who received ERT were enrolled. The study used the Altus Care™ cellular-phone-based application, which incorporated an algorithm-based approach to offer random dosing regimens within a pre-defined range set by the physician. The app enabled personalized therapeutic regimens with variations in dosages and administration times. **Results:** The second-generation AI-based personalized regimen was associated with stable responses to ERT in patients with GD1. The SF-36 quality of life scores improved in one patient, and the sense of change in health improved in two; platelet levels increased in two patients, and hemoglobin remained stable. The system demonstrated a high engagement rate among patients and caregivers, showing compliance with the treatment regimen. **Conclusions:** This feasibility study highlights the potential of using variability-based regimens to enhance ERT effectiveness in GD and calls for further and longer trials to validate these findings.

## 1. Introduction

Gaucher Disease type 1 (GD1) is a lysosomal storage disorder that affects many systems. It is an autosomal recessive disease caused by bi-allelic mutations in the glucocerebrosidase gene (*GBA1*), leading to deficiency in the enzymatic activity of the β-glucocerebrosidase and, therefore, to the lysosomal accumulation of its substrate glucosylceramide; this occurs most prominently in macrophages in the visceral tissues liver, spleen, and bone marrow, inducing a pleiotropic array of signs and symptoms, including hepatosplenomegaly, pancytopenia, and bone complications (such as bone crises, avascular necrosis, osteoporosis, and pathologic fractures) [1,2]. Glucosylsphingosine (Lyso-Gb1) is a highly sensitive and specific biomarker for diagnosing and monitoring patients with GD [3,4,5,6].

Since 1991, intravenous enzyme replacement therapy (ERT) has become the standard of care, improving almost all GD-related features, improving anemia and thrombocytopenia, reducing organomegaly (liver and spleen), and alleviating skeletal findings [5,6,7]. Therapy goals include hematological, visceral, and bone manifestations and improvement in quality of life, fatigue, and social participation [8].

Circadian rhythms and biological signaling occur in a complex network with cyclical 24 h period interactions (chronobiology) between the central and autonomic nervous systems, the endocrine glands, and the immune system. Among the factors influencing host response, those associated with circadian disruptions are emerging. Harnessing host rhythms or disrupting the immune rhythms could be exploited for clinical benefit [9]. The orchestrated molecular oscillations control the rhythmicity of numerous body events and determine the physiology of multiple metabolic and inflammatory pathways, which may be associated with the pathogenesis of GD [10,11].

Inherent intra- and inter-patient variability patterns are described in many biological systems [12,13,14,15,16,17,18,19,20,21,22,23,24,25]. Examples are variability at the DNA level, heart rate variability, and respiratory and gate variability [26,27,28,29,30]. The loss or change in physiologic variability is associated with poor prognosis [31,32,33]. The response to many drugs describes high degrees of intra- and inter-patient variability. The significant intra- and inter-patient variabilities in drug pharmacodynamics are associated with patients losing their response to drugs [34,35,36,37,38]. A constant daily dose, or a continuous increase in a dose, is more likely to be associated with drug tolerance or resistance, thus losing the clinical impact, compared with the irregular taking of the same dose or altering the daily dose [39]. Treatment regimens based on aperiodic routines of taking the medication at irregular intervals and irregular strengths were suggested to improve effects [39,40,41,42,43,44].

The constrained disorder principle (CDP) defines every biological system based on its inherent variability, bounded by dynamic boundaries [45]. This principle provides a method for platforms designed to improve response to chronic medications. CDP-based second-generation artificial intelligence systems (AI) were developed to introduce variability into treatment regimens, enhance the response to chronic medication, and address the partial or complete loss of drug efficacy [39,42,43,44]. These systems also assist in early diagnosing and monitoring patients with rare diseases [46].

The present study aimed to determine the feasibility of using an app that alters the regularity of the intervals between ERT dosages while keeping them within a range defined by the physician.

## 2. Methods

**Study Design:** The clinical trial was an open-label, prospective, single-center proof-of-concept study lasting 6 months. It aimed to assess the feasibility of using an algorithm-based regimen to improve the response to ERT in patients with GD. The trial enrolled subjects at the Gaucher Unit of the Shaare Zedek Medical Center in Jerusalem, Israel. The Shaare Zedek IRB committee approved the trial. The trial was registered at the NIH GOV No NCT06050967.

**Study Population:** Eligible subjects included males and females aged 18–75 diagnosed with GD1 and receiving at-home intravenous ERT every two weeks for six months at a regular dose of 30–60 U/kg per month.

**Inclusion and exclusion criteria:** Adult non-pregnant patients diagnosed with GD1who have been treated with ERT for at least 3 years, with no change in dosage in the last six months, were included in the study. Patients with severe infectious or malignant, autoimmune, or other disabling systemic diseases were excluded. Additionally, patients who could not provide written informed consent, had no smartphone, or did not adhere to the visit schedule and protocol were excluded.

**Second-generation AI system:** Altus Care™ is a mobile phone-based application and a product of Area9 Innovation Apps, a part of Area9 Group. It allows for the easy digitization of treatment plans or research protocols and their remote implementation. The present study utilized an algorithmic approach providing random drug dosing regimens (Oberon Sciences, Israel). The platform has been combined with treatment algorithms that use second-generation AI to provide random alterations in the dosing and times of administration of medications within a pre-defined range. It also serves as a reminder for patients to take their medications. The app provides a personalized therapeutic regimen, creating variability in dosages and administration times within physicians’ pre-defined ranges.

**Study Design:** Five participants who met the specified criteria were included in the study. All participants underwent a clinical examination during screening to ensure they met the requirements. Baseline clinical and laboratory parameters obtained at the screening included a physical exam, complete blood count (CBC), Lyso-GB1, and a 36-item short-form survey (SF-36) for quality-of-life assessment. After providing informed consent, the Altus Care™ application was installed on patients’ mobile phones.

In collaboration with the patients’ treating physicians and the home-treating nurse, an individualized treatment plan was developed for each patient within a pre-defined range of minimum and maximum weeks of ERT dosages and timing frames for its administration. According to the protocol, the patients’ monthly doses were not changed, but each dose and the timing of administration were changed randomly using the app.

As part of the study, each patient was randomly assigned a dosage between 400 units and their maximum monthly dosage. The administration time was also randomized, varying between 10 and 18 days instead of the standard 14-day interval. On the day of medication administration, patients were randomly assigned to a specific time within a few-hour window, from 7 a.m. to 2 p.m.

**Follow-up parameters:** During the follow-up period, the research coordinator conducted weekly check-ins with the patients over the phone to inquire about their clinical well-being and adherence to the treatment plan. Additionally, a physical examination, CBC, and Lyso-GB1 assessment were performed approximately every two months (twice during the study and once at the end of the follow-up) to evaluate the response to therapy. The patients completed SF-36 questionnaires at the beginning and end of the follow-up. The SF-36 is a 36-item patient-reported questionnaire that covers eight health domains: physical functioning (10 items), bodily pain (2 items), role limitations due to physical health problems (4 items), role limitations due to personal or emotional problems (4 items), emotional well-being (5 items), social functioning (2 items), energy/fatigue (4 items), and general health perceptions (5 items). Scores for each domain range from 0 to 100. A higher score indicates a more favorable health state.

**Statistics:** The Wilcoxon signed-rank test was used to analyze paired, continuous, and non-parametric data.

## 3. Results

### 3.1. Subjects’ Demographics and Safety Measures

Table 1 presents the demographics of the patients enrolled in the study. Five patients were enrolled, three males and two females, with a median age of 57.4 years. All patients completed the 6-month follow-up period and experienced no significant adverse events (AEs). Table 2 provides an example of drug administration based on the randomization algorithm.

### 3.2. Introducing Variability in Dosing and Administration Times Was Associated with Stable Clinical Outcomes of Patients with GD

**Figure 1:**
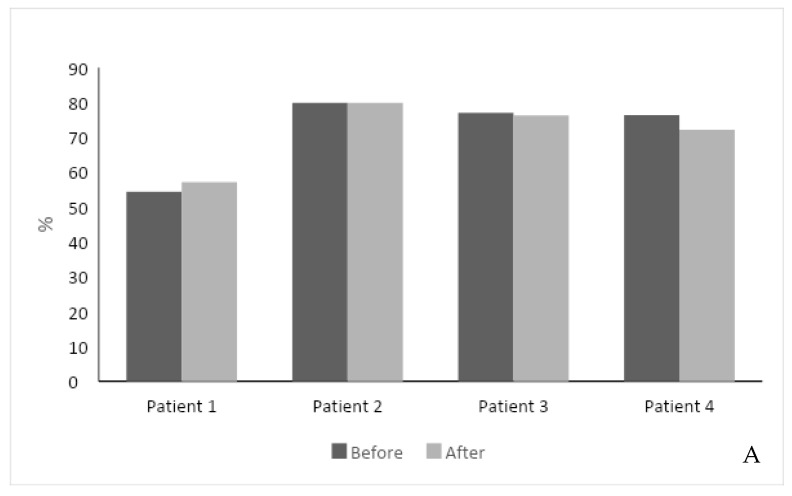

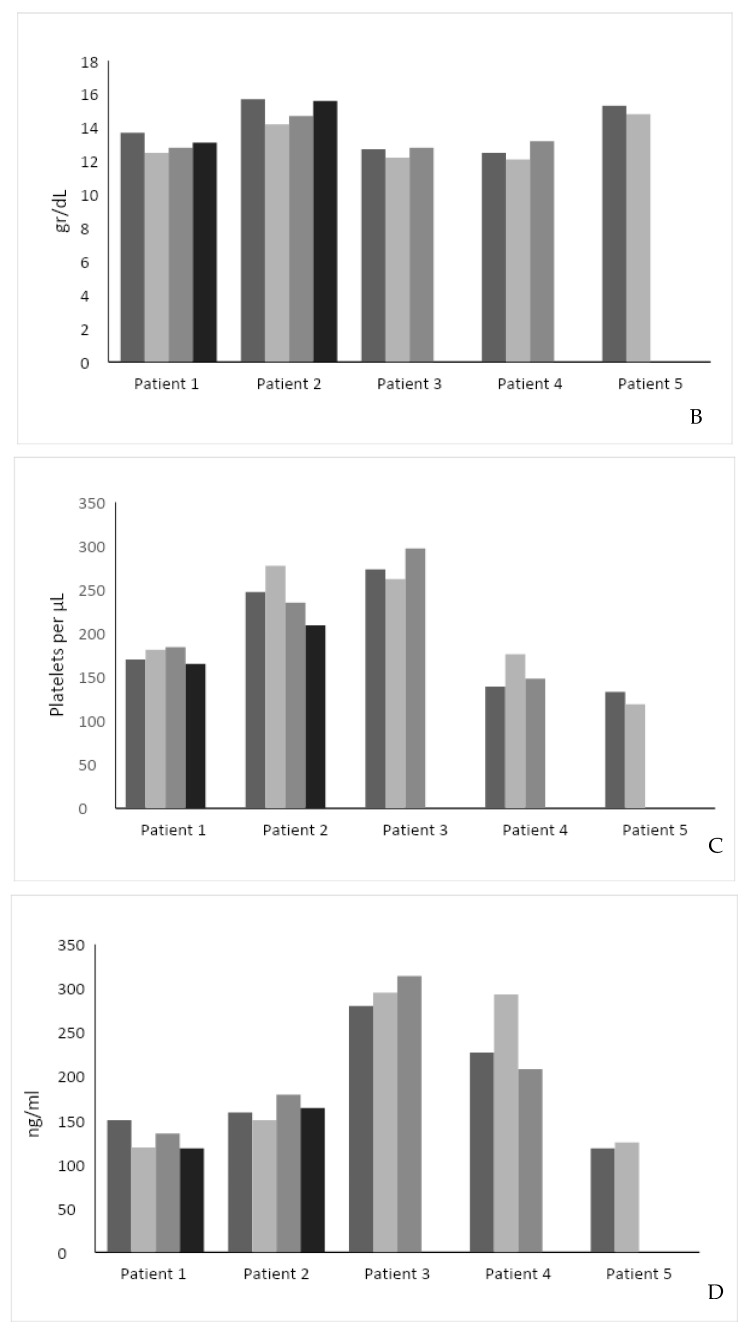
(**A**) Effect of intervention on SF-36 score. The SF-36 score was measured in 4 out of 5 patients before and after the intervention. (**B**) Effect of intervention on hemoglobin level. (**C**) Effect of intervention on serum platelet levels. (**D**) Effect of intervention on Lyso-Gb1 serum levels. Each color represents a patient follow-up encounter. Thus, the leftmost column (dark gray) represents recruitment for the study, the middle columns (lighter gray) represent two follow-up encounters during the study (2 months and 4 months post recruitment), and the rightmost column (black) represents study completion after 6 months.

The second-generation, AI-based regimen was associated with a stable response to ERT. The total SF-36 score improved in one patient with a 3% increase, did not change in three patients, and decreased by 4% in one patient. The feeling of a health change section increased in two patients (Figure 1A). One patient did not fill the SF-36 score due to his Parkinson’s disease and technical difficulty.

The hemoglobin level was stable in four patients and increased by 0.7 in one patient (Figure 1B). The median hemoglobin level was 13.7 g/dL before and 13.2 g/dL after the study intervention (*p* = NS). Platelet level increased in two patients, decreased in two patients, and was stable in one. The median platelet level was 170 × 10^9^/L before and 165 × 10^9^/L after the study intervention (*p* = 0.81) (Figure 1C).

Compared to the pre-intervention Lyso-GB1, the intervention was associated with a decrease in two patients (mean reduction: 25 ng/mL), an increase in one (34 ng/mL), and a stable level in two. The median Lyso-GB1 level was 159 ng/mL before and 164 ng/mL after the study intervention (*p* = NS) (Figure 1D).

## 4. Discussion

The results of this open-label proof-of-concept feasibility clinical trial suggest that using a second-generation personalized AI algorithm to randomize ERT regimens is both feasible and safe. Two patients experienced an increase in their platelet levels, two had a decrease in their Lyso-Gb1 levels, and two reported an improvement in their overall health. Unlike first-generation AI systems, the outcome-based second-generation system showed a high engagement rate among patients and physicians. Patients, treating physicians, and home ERT nurses all used the app and followed the treatment regimen as instructed.

The constrained disorder principle (CDP) defines every biological system based on its inherent variability, bounded by dynamic boundaries [45]. According to the CDP, systems adapt to their noisy environments by adjusting their level of intrinsic variability. The CDP sees system malfunctions as resulting from either too little or too much variability. It offers a method for addressing complex system malfunctions by modifying the levels of variability.

Based on this principle, disease or loss of response to medication reflects a reduced degree of variability or an increased variability beyond borders [41,45,47,48]. CDP-based second-generation AI systems are designed to implement variability signatures to improve response to chronic therapies or overcome the loss of response to chronic drugs. This platform can improve the response to therapeutic regimens in chronic diseases by introducing variability in administration times and dosages within approved ranges [46,49,50,51,52,53,54,55,56,57,58,59,60,61,62,63,64,65,66,67].

Over 10% of patients with GD demonstrate an incomplete response to ERT or develop partial loss of response with time, a significant health problem for the patients and their caregivers [2,68,69,70,71,72]. Several mechanisms contribute to the loss of response to ERT. In some patients, the immune system may recognize the synthetic enzyme used in ERT as a foreign substance, producing neutralizing antibodies [73,74]. These antibodies can render the enzyme less effective or ineffective, reducing the therapeutic benefit. GD is a progressive disorder, and despite ERT, the accumulation of glucocerebroside can worsen over time. As the disease advances, the symptoms may become more severe, leading to the perception of a loss of response to therapy. The effectiveness of ERT is impacted by patient compliance with the treatment regimen. Missing doses or a lack of adherence to the recommended schedule can lead to suboptimal results. Other medical conditions or medications a patient takes can interfere with ERT’s effectiveness. When a loss of response to ERT is observed, physicians may need to explore alternative treatment options, such as switching to a different ERT or considering other therapeutic approaches like substrate reduction therapy or chaperone therapy [75]. In addition, the continuously dynamic inter- and intra-patient variability in response to ERT is a significant intervention challenge.

CDP-based second-generation AI system provides a tool that can dynamically and constantly tailor the regimen to biological variability in a personalized way [42]. Using biological noise and chronobiology-based algorithms is designed to overcome tolerance and partial or complete loss of response to chronic therapies in patients suffering from chronic diseases. The use of this platform was shown to have a beneficial clinical effect on multiple chronic diseases, including chronic heart failure, chronic pain, and multiple sclerosis. In patients with heart failure who developed diuretic resistance, using a CDP-based second-generation AI app that introduced variability into treatment regimens improved patients’ clinical and laboratory parameters. Using the system reduced hospitalization and emergency room admissions, showing a high rate of engagement with the system by patients and caregivers [76]. Similar beneficial data were demonstrated in patients with chronic pain and multiple sclerosis [48].

In the present study, the first level of the algorithm, an open-loop system, was used. It involves randomizing dose and administration times within a pre-defined approved range without AI. The clinical outcome did not affect the randomization.

The second level of the algorithm tailors variability to outcomes based on clinical and laboratory data collected from the subject. The third level involves quantifying signatures of variability in a personalized way, such as heart rate variability, alterations in cytokine secretion, and other disease biomarkers, and implementing them into the treatment algorithms [39,41,42,43,44].

The limitations of our study are the small sample size and the relatively short duration of intervention that may not have been sufficient to observe significant changes in response to ERT, the lack of diversity in this limited patient population, a lack of a control group, the open-labeled design, and the conduction in a single-center; there were non-significant changes in hemoglobin levels and platelet counts in some of the patients. In addition, the SF-36 quality of life score is self-reported and may be subject to individual interpretation and reporting biases.

More extended observation periods are expected to achieve an effect. In addition, it has been recently suggested that GD-specific patient-reported outcome measures (PROM) may be more relevant to showing small, meaningful changes, and these should be used in the subsequent study [7,8].

Collaboration with numerous experts is crucial for successfully integrating AI into healthcare. It ensures that AI systems are trained on precise, high-quality data and that their outputs are clinically relevant and safe for patient care. This is fundamental in advancing personalized medicine and improving patient outcomes [68].

In summary, CDP-based second-generation AI systems in patients with GD may improve their ERT response. More extensive and longer clinical trials with larger numbers of patients who have shown poor reactions to their medication are necessary in understanding better how variability-based regimens can be utilized in these patients.

## Figures and Tables

**Table 1:** Baseline characteristics.

Age, median (range)		57.4 (37–65)
Male, number (%)		3 (60)
Time since on Enzyme replacement therapy, median, Years		26.8
High Lyso-GB1 levels		2 (40%)
Gaucher disease complications	Anemia	0
	Thrombocytopenia	2 (40%)
	Bone pain/osteonecrosis	1 (20%)
	s/p splenectomy	2 (40%)
Concomitant diseases	Parkinson’s disease (%)	1 (20)

**Table 2:** An example of drug administration based on the randomization algorithm for one of the subjects in the study.

Administration time: Days since recruitment	1	14	31	45	62	74	91	104	121	135	149	165
Dosage	3200	2000	4400	800	1200	4000	800	4400	3600	1600	2000	3200
Administration time—hour	8:30	9:45	8:45	9:00	9:30	8:15	7:15	7:00	9:00	7:45	9:00	8:45

## Data Availability

Data available on request due to restrictions eg privacy or ethical.

## Abbreviations

GD1, Gaucher Disease 1; Lyso-Gb1, glucosylsphingosine; ERT, enzyme replacement therapy; AI, artificial intelligence; CBC, complete blood count; SF-36, 36-item short-form survey; CDP, constrained disorder principle; PROM, patient-reported outcome measures (PROM).

## References

[1] Rolfs A., Giese A.-K., Grittner U., Mascher D., Elstein D., Zimran A., Böttcher T., Lukas J., Hübner R., Gölnitz U. (2018). How we manage Gaucher Disease in the era of choices. Br. J. Haematol..

[2] Elstein D., Mellgard B., Dinh Q., Lan L., Qiu Y., Cozma C., Eichler S., Böttcher T., Zimran A. (2018). Recent advances in the diagnosis and management of Gaucher disease. Expert Rev. Endocrinol. Metab..

[3] Zimran A., Elstein D., Gonzalez D.E., Lukina E.A., Qin Y., Dinh Q., Turkia H.B. (2013). Glucosylsphingosine is a highly sensitive and specific biomarker for primary diagnostic and follow-up monitoring in Gaucher disease in a non-Jewish, Caucasian cohort of Gaucher disease patients. PLoS ONE.

[4] Elstein D., Mellgard B., Dinh Q., Lan L., Qiu Y., Cozma C., Eichler S., Böttcher T., Zimran A. (2017). Reductions in glucosylsphingosine (lyso-Gb1) in treatment-naïve and previously treated patients receiving velaglucerase alfa for type 1 Gaucher disease: Data from phase 3 clinical trials. Mol. Genet. Metab..

[5] Zimran A., Elstein D., Gonzalez D.E., Lukina E.A., Qin Y., Dinh Q., Turkia H.B. (2018). Treatment-naive Gaucher disease patients achieve therapeutic goals and normalization with velaglucerase alfa by 4years in phase 3 trials. Blood Cells Mol. Dis..

[6] Elstein D., Mehta A., Hughes D.A., Giraldo P., Charrow J., Smith L., Shankar S.P., Hangartner T.N., Kunes Y., Wang N. (2015). Safety and efficacy results of switch from imiglucerase to velaglucerase alfa treatment in patients with type 1 Gaucher disease. Am. J. Hematol..

[7] Elstein D., Zimran A. (2009). Review of the safety and efficacy of imiglucerase treatment of Gaucher disease. Biologics.

[8] Biegstraaten M., Cox T.M., Belmatoug N., Berger M.G., Collin-Histed T., Vom Dahl S., Di Rocco M., Fraga C., Giona F., Giraldo P. (2018). Management goals for type 1 Gaucher disease: An expert consensus document from the European working group on Gaucher disease. Blood Cells Mol. Dis..

[9] Westwood M.L., O’donnell A.J., de Bekker C., Lively C.M., Zuk M., Reece S.E. (2019). The evolutionary ecology of circadian rhythms in infection. Nat. Ecol. Evol..

[10] Mukherjee S., Maitra S.K. (2015). Gut Melatonin in Vertebrates: Chronobiology and Physiology. Front. Endocrinol..

[11] Haus E. (2002). Chronobiology of the mammalian response to ionizing radiation. Potential applications in oncology. Chronobiol. Int..

[12] Ilan Y. (2019). Overcoming randomness does not rule out the importance of inherent randomness for functionality. J. Biosci..

[13] Ilan Y. (2019). Generating randomness: Making the most out of disordering a false order into a real one. J. Transl. Med..

[14] Ilan Y. (2020). Advanced Tailored Randomness: A Novel Approach for Improving the Efficacy of Biologica l Systems. J. Comput. Biol..

[15] Ilan Y. (2020). Order Through Disorder: The Characteristic Variability of Systems. Front Cell Dev. Biol..

[16] El-Haj M., Kanovitch D., Ilan Y. (2019). Personalized inherent randomness of the immune system is manifested by an individualized response to immune triggers and immunomodulatory therapies: A novel platform for designing personalized immunotherapies. Immunol. Res..

[17] Ilan Y. (2019). Randomness in microtubule dynamics: An error that requires correction or an inherent plasticity required for normal cellular function?. Cell Biol. Int..

[18] Ilan Y. (2019). Microtubules: From understanding their dynamics to using them as potential therapeutic targets. J. Cell Physiol..

[19] Ilan-Ber T., Ilan Y. (2019). The role of microtubules in the immune system and as potential targets for gut-based immunotherapy. Mol. Immunol..

[20] Forkosh E., Kenig A., Ilan Y. (2020). Introducing variability in targeting the microtubules: Review of current mechanisms and future directions in colchicine therapy. Pharmacol. Res. Perspect..

[21] Ilan Y. (2019). beta-Glycosphingolipids as Mediators of Both Inflammation and Immune Tolerance: A Manifestation of Randomness in Biological Systems. Front. Immunol..

[22] Ilan Y. (2022). Microtubules as a potential platform for energy transfer in biological systems: A target for implementing individualized, dynamic variability patterns to improve organ function. Mol. Cell. Biochem..

[23] Ilan Y. (2022). Enhancing the plasticity, proper function and efficient use of energy of the Sun, genes and microtubules using variability. Clin. Transl. Discov..

[24] Shabat Y., Lichtenstein Y., Ilan Y. (2021). Short-Term Cohousing of Sick with Healthy or Treated Mice Alleviates the Inflammatory Response and Liver Damage. Inflammation.

[25] Goldberger A.L. (1996). Non-linear dynamics for clinicians: Chaos theory, fractals, and complexity at the bedside. Lancet.

[26] Finn E.H., Misteli T. (2019). Molecular basis and biological function of variability in spatial genome organization. Science.

[27] Shields R.W. (2009). Heart rate variability with deep breathing as a clinical test of cardiovagal function. Cleve. Clin. J. Med..

[28] Kox M., Pompe J.C., van der Hoeven J.G., Hoedemaekers C.W., Pickkers P. (2011). Influence of different breathing patterns on heart rate variability indices and reproducibility during experimental endotoxaemia in human subjects. Clin. Sci..

[29] König N., Singh N.B., Baumann C.R., Taylor W.R. (2016). Can Gait Signatures Provide Quantitative Measures for Aiding Clinical Decision-Making? A Systematic Meta-Analysis of Gait Variability Behavior in Patients with Parkinson’s Disease. Front. Hum. Neurosci..

[30] Singh N., Moneghetti K.J., Christle J., Hadley D., Plews D., Froelicher V. (2018). Heart Rate Variability: An Old Metric with New Meaning in the Era of using mHealth Technologies for Health and Exercise Training Guidance. Part One: Physiology and Methods. Arrhythm. Electrophysiol. Rev..

[31] Nayyar S., Hasan M.A., Roberts-Thomson K.C., Sullivan T., Baumert M. (2017). Effect of Loss of Heart Rate Variability on T-Wave Heterogeneity and QT Variability in Heart Failure Patients: Implications in Ventricular Arrhythmogenesis. Cardiovasc. Eng. Technol..

[32] Avolio A. (2013). Heart rate variability and stroke: Strange attractors with loss of complexity. J. Hypertens..

[33] Moon Y., Sung J., An R., Hernandez M.E., Sosnoff J.J. (2016). Gait variability in people with neurological disorders: A systematic review and meta-analysis. Hum. Mov. Sci..

[34] Leino A.D., King E.C., Jiang W., Vinks A.A., Klawitter J., Christians U., Woodle E.S., Alloway R.R., Rohan J.M. (2018). Assessment of tacrolimus intrapatient variability in stable adherent transplant recipients: Establishing baseline values. Am. J. Transplant..

[35] Gueta I., Markovits N., Yarden-Bilavsky H., Raichlin E., Freimark D., Lavee J., Loebstein R., Peled Y. (2018). High tacrolimus trough level variability is associated with rejections after heart transplant. Am. J. Transplant..

[36] Gueta I., Markovits N., Yarden-Bilavsky H., Raichlin E., Freimark D., Lavee J., Loebstein R., Peled Y. (2018). Intrapatient variability in tacrolimus trough levels after solid organ transplantation varies at different postoperative time periods. Am. J. Transplant..

[37] Del Bello A., Congy-Jolivet N., Danjoux M., Muscari F., Lavayssière L., Esposito L., Hebral A.-L., Bellière J., Kamar N. (2018). High tacrolimus intra-patient variability is associated with graft rejection, and de novo donor-specific antibodies occurrence after liver transplantation. World J. Gastroenterol..

[38] Niederer S.A., Lumens J., Trayanova N.A. (2019). Computational models in cardiology. Nat. Rev. Cardiol..

[39] Ilan Y. (2020). Overcoming Compensatory Mechanisms toward Chronic Drug Administration to Ensure Long-Term, Sustainable Beneficial Effects. Mol. Ther. Methods Clin. Dev..

[40] Kyriazis M. (2003). Practical applications of chaos theory to the modulation of human ageing: Nature prefers chaos to regularity. Biogerontology.

[41] Ilan Y. (2023). Making use of noise in biological systems. Prog. Biophys. Mol. Biol..

[42] Ilan Y. (2020). Second-Generation Digital Health Platforms: Placing the Patient at the Center and Focusing on Clinical Outcomes. Front. Digit. Health.

[43] Ilan Y. (2021). Improving Global Healthcare and Reducing Costs Using Second-Generation Artificial Intelligence-Based Digital Pills: A Market Disruptor. Int. J. Environ. Res. Public Health.

[44] Ilan Y. (2022). Next-Generation Personalized Medicine: Implementation of Variability Patterns for Overcoming Drug Resistance in Chronic Diseases. J. Pers. Med..

[45] Ilan Y. (2022). The constrained disorder principle defines living organisms and provides a method for correcting disturbed biological systems. Comput. Struct. Biotechnol. J..

[46] Hurvitz N., Azmanov H., Kesler A., Ilan Y. (2021). Establishing a second-generation artificial intelligence-based system for improving diagnosis, treatment, and monitoring of patients with rare diseases. Eur. J. Hum. Genet..

[47] Ilan Y. (2023). Constrained disorder principle-based variability is fundamental for biological processes: Beyond biological relativity and physiological regulatory networks. Prog. Biophys. Mol. Biol..

[48] Sigawi T., Lehmann H., Hurvitz N., Ilan Y. (2023). Constrained Disorder Principle-Based Second-Generation Algorithms Implement Quantified Variability Signatures to Improve the Function of Complex Systems. J. Bioinform. Syst. Biol..

[49] Kessler A., Weksler-Zangen S., Ilan Y. (2020). Role of the Immune System and the Circadian Rhythm in the Pathogenesis of Chronic Pancreatitis: Establishing a Personalized Signature for Improving the Effect of Immunotherapies for Chronic Pancreatitis. Pancreas.

[50] Ishay Y., Kolben Y., Kessler A., Ilan Y. (2021). Role of circadian rhythm and autonomic nervous system in liver function: A hypothetical basis for improving the management of hepatic encephalopathy. Am. J. Physiol. Liver Physiol..

[51] Kolben Y., Weksler-Zangen S., Ilan Y. (2021). Adropin as a potential mediator of the metabolic system-autonomic nervous system-chronobiology axis: Implementing a personalized signature-based platform for chronotherapy. Obes. Rev..

[52] Kenig A., Kolben Y., Asleh R., Amir O., Ilan Y. (2021). Improving Diuretic Response in Heart Failure by Implementing a Patient-Tailored Variability and Chronotherapy-Guided Algorithm. Front. Cardiovasc. Med..

[53] Azmanov H., Ross E.L., Ilan Y. (2021). Establishment of an Individualized Chronotherapy, Autonomic Nervous System, and Variability-Based Dynamic Platform for Overcoming the Loss of Response to Analgesics. Pain Physician.

[54] Potruch A., Khoury S.T., Ilan Y. (2020). The role of chronobiology in drug-resistance epilepsy: The potential use of a variability and chronotherapy-based individualized platform for improving the response to anti-seizure drugs. Seizure.

[55] Isahy Y., Ilan Y. (2021). Improving the long-term response to antidepressants by establishing an individualized platform based on variability and chronotherapy. Int. J. Clin. Pharmacol. Ther..

[56] Khoury T., Ilan Y. (2019). Introducing Patterns of Variability for Overcoming Compensatory Adaptation of the Immune System to Immunomodulatory Agents: A Novel Method for Improving Clinical Response to Anti-TNF Therapies. Front. Immunol..

[57] Khoury T., Ilan Y. (2021). Platform introducing individually tailored variability in nerve stimulations and dietary regimen to prevent weight regain following weight loss in patients with obesity. Obes. Res. Clin. Pract..

[58] Kenig A., Ilan Y. (2019). A Personalized Signature and Chronotherapy-Based Platform for Improving the Efficacy of Sepsis Treatment. Front. Physiol..

[59] Ilan Y. (2019). Why targeting the microbiome is not so successful: Can randomness overcome the adaptation that occurs following gut manipulation?. Clin. Exp. Gastroenterol..

[60] Gelman R., Bayatra A., Kessler A., Schwartz A., Ilan Y. (2020). Targeting SARS-CoV-2 receptors as a means for reducing infectivity and improving antiviral and immune response: An algorithm-based method for overcoming resistance to antiviral agents. Emerg. Microbes Infect.

[61] Ishay Y., Potruch A., Schwartz A., Berg M., Jamil K., Agus S., Ilan Y. (2021). A digital health platform for assisting the diagnosis and monitoring of COVID-19 progression: An adjuvant approach for augmenting the antiviral response and mitigating the immune-mediated target organ damage. Biomed. Pharmacother..

[62] Ilan Y., Spigelman Z. (2020). Establishing patient-tailored variability-based paradigms for anti-cancer therapy: Using the inherent trajectories which underlie cancer for overcoming drug resistance. Cancer Treat Res. Commun..

[63] Ilan Y. (2021). Digital Medical Cannabis as Market Differentiator: Second-Generation Artificial Intelligence Systems to Improve response. Front. Med..

[64] Gelman R., Berg M., Ilan Y. (2022). A Subject-Tailored Variability-Based Platform for Overcoming the Plateau Effect in Sports Training: A Narrative Review. Int. J. Environ. Res. Public Health.

[65] Azmanov H., Bayatra A., Ilan Y. (2022). Digital Analgesic Comprising a Second-Generation Digital Health System: Increasing Effectiveness by Optimizing the Dosing and Minimizing Side Effects. J. Pain Res..

[66] Hurvitz N., Elkhateeb N., Sigawi T., Rinsky-Halivni L., Ilan Y. (2022). Improving the effectiveness of anti-aging modalities by using the constrained disorder principle-based management algorithms. Front. Aging.

[67] Kolben Y., Azmanov H., Gelman R., Dror D., Ilan Y. (2023). Using chronobiology-based second-generation artificial intelligence digital system for overcoming antimicrobial drug resistance in chronic infections. Ann. Med..

[68] Gupta P., Pastores G. (2018). Pharmacological treatment of pediatric Gaucher disease. Expert Rev Clin Pharmacol.

[69] Charrow J., Scott C.R. (2015). Long-term treatment outcomes in Gaucher disease. Am. J. Hematol..

[70] Bennett L.L., Mohan D. (2013). Gaucher disease and its treatment options. Ann. Pharmacother..

[71] Zimran A., Ilan Y., Elstein D. (2009). Enzyme replacement therapy for mild patients with Gaucher disease. Am. J. Hematol..

[72] Ilan Y., Elstein D., Zimran A. (2009). Glucocerebroside: An evolutionary advantage for patients with Gaucher disease and a new immunomodulatory agent. Immunol. Cell Biol..

[73] Zhao H., Bailey L.A., Grabowski G.A. (2003). Enzyme therapy of gaucher disease: Clinical and biochemical changes during production of and tolerization for neutralizing antibodies✩ ✩This study was supported by NIH Grant R01 DK 36729 to GAG. Blood Cells Mol. Dis..

[74] Wang J., Lozier J., Johnson G., Kirshner S., Verthelyi D., Pariser A., Shores E., Rosenberg A. (2008). Neutralizing antibodies to therapeutic enzymes: Considerations for testing, prevention and treatment. Nat. Biotechnol..

[75] Zimran A. (2011). How I treat Gaucher disease. Blood J. Am. Soc. Hematol..

[76] Gelman R., Hurvitz N., Nesserat R., Kolben Y., Nachman D., Jamil K., Agus S., Asleh R., Amir O., Berg M. (2023). A second-generation artificial intelligence-based therapeutic regimen improves diuretic resistance in heart failure: Results of a feasibility open-labeled clinical trial. Biomed. Pharmacother..

